# Acute erythroid leukemia leading to the diagnosis of Schwachman-Diamond syndrome

**DOI:** 10.1007/s44313-024-00008-8

**Published:** 2024-03-06

**Authors:** Bernhard Strasser, Sebastian Mustafa, Josef Tomasits, Alexander Haushofer

**Affiliations:** 1 Institute of Clinical Chemistry and Laboratory Medicine, Klinikum Wels-Grieskirchen, Grieskirchnerstraße 42, 4600 Wels, Austria; 2 Institute of Clinical Chemistry and Laboratory Medicine, Kepler University Hospital, Linz, Austria

**Keywords:** Schwachman-Diamond syndrome, Acute erythroid leukemia, SBDS mutation


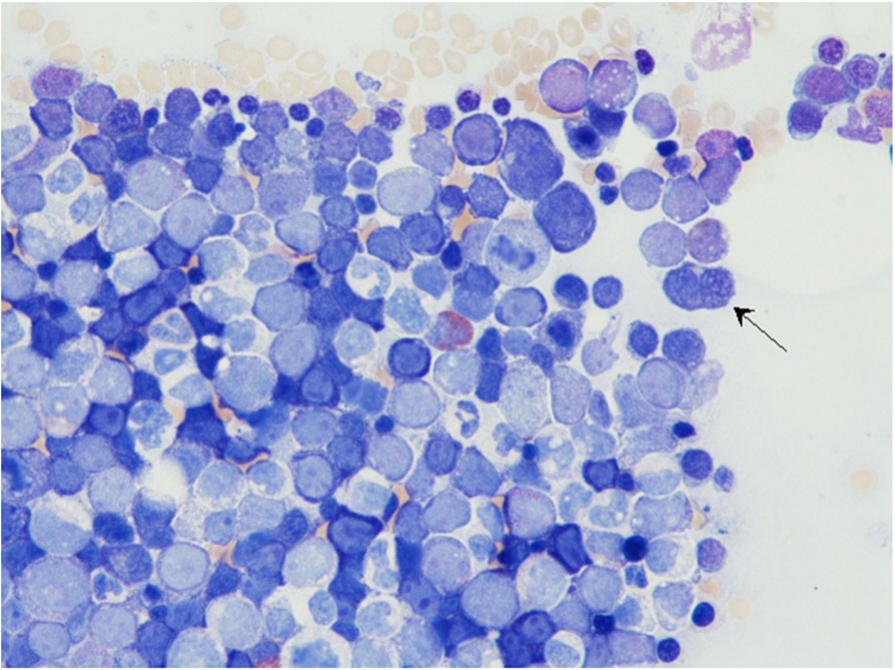
A bone marrow puncture was performed on a 38-year-old female patient with severe pancytopenia and leukoerythroblastosis (two blasts and five erythropoietic precursor cells). A cytomorphological investigation established the diagnosis of acute myeloid leukemia of the acute erythroid leukemia subtype. Myeloblasts accounted for 6% and erythropoietic cells accounted for 83% with a strong dominance of proerythroblasts, whereas mature hematopoietic cells were mostly absent. The proerythroblasts showed finely dispersed nuclei and minimal agranular cytoplasm. In addition, double nuclearity of erythropoietic precursor cells was frequently observed (indicated by the arrow). Immuncytological investigation provided a CD117+/71++/MPO- profile. The neoplastic cells repeatedly showed vacuolization, which is typically associated with TP53 mutation. In this patient, two somatic mutations, TP53 c.711G>A and c.706T>A, and a complex karyotype supported the diagnosis of acute erythroid leukemia.

The young age of the patient was atypical for a diagnosis of acute erythroid leukemia. Additional comorbidities included severe exocrine pancreas insufficiency. The patient exhibited growth restriction and microcephaly. A syndrome of germline predisposition to hematological neoplasms was suspected, primarily Schwachman-Diamond syndrome. Targeted sequencing of the SBDS gene revealed two heterozygote mutations, c.183_184delinsCT and c.258+2T>C, which confirmed the diagnosis of Schwachman-Diamond syndrome.

